# Application of Multi-Perspectives in Tea Breeding and the Main Directions

**DOI:** 10.3390/ijms241612643

**Published:** 2023-08-10

**Authors:** Haozhen Li, Kangkang Song, Xiaohua Zhang, Di Wang, Shaolin Dong, Ying Liu, Long Yang

**Affiliations:** College of Plant Protection and Agricultural Big-Data Research Center, Shandong Agricultural University, Tai’an 271018, China

**Keywords:** tea, breeding, omics, germplasm, applications

## Abstract

Tea plants are an economically important crop and conducting research on tea breeding contributes to enhancing the yield and quality of tea leaves as well as breeding traits that satisfy the requirements of the public. This study reviews the current status of tea plants germplasm resources and their utilization, which has provided genetic material for the application of multi-omics, including genomics and transcriptomics in breeding. Various molecular markers for breeding were designed based on multi-omics, and available approaches in the direction of high yield, quality and resistance in tea plants breeding are proposed. Additionally, future breeding of tea plants based on single-cellomics, pangenomics, plant–microbe interactions and epigenetics are proposed and provided as references. This study aims to provide inspiration and guidance for advancing the development of genetic breeding in tea plants, as well as providing implications for breeding research in other crops.

## 1. Introduction

Tea plants hold important health and cultural values as a non-alcoholic beverage, one of the most widely consumed drinks globally. Originating in Southeast Asia [1], tea plants have been widely distributed around the world, with over 5 million hectares under cultivation in more than 60 tea-growing countries worldwide [2]. Nearly 70% of the world’s population consumes between 18 and 20 billion cups of tea every day [3]. Tea leaves feature characteristic secondary metabolites such as polyphenols [4], catechins [5] and caffeine, which play a crucial role in determining the yield and quality of the tea leaves. Additionally, these compounds offer numerous health benefits, including the prevention and treatment of cardiovascular disease [6] and cancer prevention, etc. [7]. Research in breeding can effectively cultivate excellent varieties of tea plants, explore target traits, and deepen the understanding of the genetic background, which could lay the foundation for the development of the tea industry.

Germplasm resources are the key in plant breeding, carrying abundant genetic resources [8]. Germplasm resources, including both main and wild varieties, represent the potential source of plant genetic diversity and they also contain potential alleles [9]. Allelic variation in key biological pathways facilitate the achievement of crop breeding goals [10]. The germplasm resources that have been developed by natural selection and domestication of tea plants over the long-term provide a gene pool for breeding target varieties., and the development of omics promotes the utilization of tea plants resources with high value traits or excellent quality. Omics serve as a powerful tool for studying the function, structure and genetic information of the plant body, which emerged as an essential strategy for modern plant breeding [11]. For example, transcriptome sequencing emerged as a prominent method for functional analysis and gene prediction [12], playing a crucial role in studying the mechanisms of regulation [13]. The metabolome was adapted to detect a wide range of metabolites, enabling the exploration of intricate biological pathways in plants [14,15,16]. The breeding goals of high yield, quality and resistance to biotic and abiotic stresses in tea plants were achieved through the identification of gene functions [17], transcriptional regulatory mechanisms [18], changes in metabolites [19], localization of genetic variation loci and dissection of complex trait mechanisms [20,21]. Marker-assisted breeding could also be achieved for target traits, and with the continued publication of sequencing data, molecular markers were designed to be applied in tea plants research for origin and evolutionary probing [22], kinship and genetic diversity, and other related analyses [23]. Furthermore, the development of tea plants breeding could not be achieved without the application of the above multi-dimensional perspectives, which in turn provided the necessary supports and insights.

Different techniques and tools have been applied to resolve biological issues such as origin, domestication, genetic structure, yield, quality, resistance and other traits, and have advanced the process of tea plants breeding. However, tea plants still suffer from slow conventional breeding rates and genetic resistance limitations in the breeding process, and their breeding work still confronts numerous challenging events. In this study, the current status and utilization of germplasm resources, the application of multi-omics in tea plants breeding, molecular markers developed, major breeding directions and approaches were the focus of the research, providing essential insights and novel directions for seeking more appropriate breeding techniques and strategies for tea plants in the future.

## 2. Status of Germplasm Resources and Utilization

Germplasm was the genetic material that was passed from one generation to the next, and germplasm resources formed through long-term natural evolution and artificial selection were the original material for tea plants breeding, as well as the material foundations for studying the origin and evolution of species [24,25]. The collection and conservation of germplasm resources were a vital aspect of tea plants breeding. China was the first country in the world to discover and utilize tea plants, which were passed around the world via the maritime and land Silk Roads, and the evolution and selection of which resulted in an abundant germplasm resource for thousands of years [26]. In contrast to the earliest wild tea plants, modern breeding orientations led to tea varieties with good taste, high yields and adaptability to exposure the different stresses.

Currently, more than 350 species of the genus *Camellia* have been recorded [27], of which two main cultivars are *Camellia sinensis* var. *sinensis* (CSS; Chinese type) and *Camellia sinensis* var. *assamica* (CSA; Assam type), which were used to produce green, black, dark, Oolong, white and yellow teas [28]. Both types of tea plants were distinctive in their type and geographical distribution, with CSA being grown mainly in very warm tropical regions, unlike the more widespread geographical location where CSS was cultivated. Environmental differences were responsible for the phenotypic differences, with CSS representing a slow-growing shrub of small leaves that could withstand cold climates, while CSA was a fast-growing species with large leaves that were sensitive to cold weather [29,30]. Unlike the drinking function, some species such as *C. oleifera*, *C. semiserrata* and *C. chekiangoleosa* were used in the production of edible oils as well as in functional foods, pharmaceuticals and beauty products [31,32]. Flowering species such as *C. reticulata*, *C. sasanqua* and *C. saluensis* were employed for ornamental purposes [33,34].

Germplasm resources could provide the source of genes for desired traits to rapidly advance tea plants breeding, and technologies such as distant hybridization, genetic engineering and cellular engineering were applied to tea plants breeding work. The breeding of tea plants hybrids was inseparable from CSS and CSA germplasm resources, in which the accumulation of tea plants quality metabolites was identified in relation to the germplasm of the F1 descendants of hybrids between high-yielding CSA varieties and high-quality CSS clones; thus, the breeding of hybrids provided more options for the selection of tea plants breeding materials [35]. In retrospect, hybridization had been an important method of breeding and refinement in tea plants breeding, and under-utilized CSA with more rare alleles and private haplotypes were considered as a worthy germplasm resource in tea plants breeding [36]. Moreover, at the genetic level, germplasm resources contained extensive alleles and the identification and application of allelic variation for genetic improvement was the essential approach to germplasm resources research. Due to the characteristics of tea plants, the genome was highly heterozygous and allelic variants have been identified, for example, allelic variants had been discovered in oolong tea related to aroma and stress tolerance traits, and marker-assisted breeding of allelic variants had broadened the pathway for enhancing stress tolerance and aroma in other tea varieties [37]. In addition, the specific expression of alleles for energy and terpene metabolic pathways from the maternal line and glutathione-rich metabolism of the paternal line were identified in oolong tea hybrids, respectively, providing options for future breeding improvement using alleles contributing to more biological functions to regulate tea plants quality [38]. The vast variations between tea plants germplasm resources offered potential scope for breeding, and the variations in Western Himalayan germplasm resources provided genotypic information for the development of asexual lines [39], and sustainable breeding could be promoted through the selection of high-quality genotypes. Albino teas usually have high ratios of theanine that were beneficial to the flavor of the tea plants and catechin indices; therefore, the germplasm resources could be efficiently bred according to their characteristics [40].

The target traits of current cultivars were mainly derived from natural and induced genetic diversity [41], and excessive use of limited germplasm resources in the breeding process will result in genetic erosion and thus, loss of genetic diversity [42]. Thus, accelerating the selection of excellent varieties and germplasm innovation was required and therefore, increased the diversity of germplasm resources and provided more sources of raw material for the sustainability of tea plants breeding. Furthermore, germplasm diversity might be achieved through the study of variety-specific mechanisms of quality-related metabolites. Assuming that the main cultivars of tea plants were subject to mutations or lacking resistance to unexpected diseases, a substantial reduction in yield or loss of quality could occur. The genetic diversity of germplasm resources was the valuable resource for breeding, and in addition to the proper utilization of existing germplasm, the conservation and transmission of resources provided the final barrier to safeguard the breeding of tea plants.

## 3. Application of Multidimensional Omics in Tea Plants Genetic Breeding

**Figure 1:**
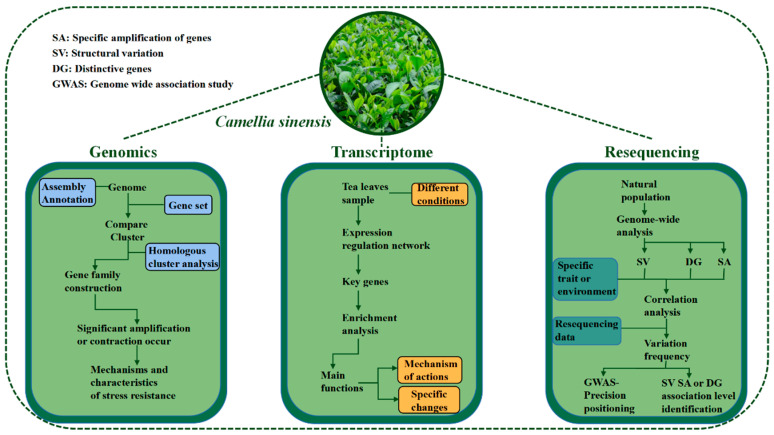
Pattern diagram of multiomics in mining abiotic stress gene resources of tea plants.

With the development of high-throughput sequencing technologies and the reduction of sequencing costs [43], the application of multidimensional genomics in tea plants genetic breeding was becoming more widespread. This section presented the current status of research on genomic, transcriptomic, metabolomic and resequencing technologies in tea plants and drew a schematic diagram of multi-omics mining of genetic resources under abiotic stress adversity (Figure 1) with a view to providing insights for researchers.

### 3.1. Genome

The release of the genome of the first model crop, Arabidopsis thaliana, kicked off a chapter in the genome era [44]. The assembly of genomes could further our understanding of the genetic basis of plant diversity [45], understand the mechanisms of adaptive evolution and ultimately accelerate the applications for breeding plants [46,47]. For tea plants, as an essential cash crop, the research in the field of genomics was of relevance. Yunkang10 was the first tea high-quality genome whose assembly revealed that enhanced catechin synthesis and stress tolerance were caused by the spectrum-specific amplification of genes related to flavonoid metabolism biosynthesis. Besides, elevated catechin and caffeine contents were dependent on high expression of the biosynthesis genes in comparison across cultivars, which also refined research on the biosynthesis of characteristic tea plants’ secondary metabolites and functional genomics [48]. The release of the draft genome sequence of tea shuchazao had identified CSS and CSA that diverged from one ancestor, discovered copies of genes that contribute to secondary metabolite synthesis caused by homologous duplication, and determined key genes for theanine synthesis, not only deepening the comprehension of enhancing tea plants quality but also setting the foundation for tea plants breeding refinement [49]. In addition, researchers constructed a chromosome-level reference genome of size 2.94 Gb by genomic and resequencing techniques and discovered that the expansion of the driving genome size was induced by insertions of LTR retrotransposons in comparison with previously published genomes, and reported that tandem repeats enabled the amplification of terpene synthase associated with tea aroma, and also confirmed the theory that cultivated tea plants originated in southwest China [22]. Meanwhile, the Longjing43 genome revealed that it underwent the same WGD event as SCZ and YK10 25 million years ago; the expansion of the gene family of germacrene D synthase (*TPSGD*) associated with secondary metabolites and *NBS-ARC* associated with resistance in genome evolution was discovered. The advantages of CSS in terms of flavor and resistance compared to CSA during domestication was obtained [50]. The successive releases of the tea plants genome (Table 1) have provided additional resources for marker-assisted breeding, as well as advancing research in the areas of genetic breeding, evolutionary origins, metabolite synthesis resolution and structural variations.

### 3.2. Transcriptome and Metabolome

Transcriptomic and metabolomic analyses have been extensively engaged to unravel the mechanisms of transcriptional regulation and metabolite changes related to characteristic secondary metabolite synthesis, stress resistance and yield traits in tea plants. Meanwhile, with the development of biotechnology, some representative genes related to the biosynthesis of secondary metabolites and abiotic and biotic stresses in tea plants that have been cloned and characterized were increasingly made available for future improvement reference (Table 2). Apart from these, some applications of omics were summarized. For example, Epigallocatechin-3-gallate (*EGCG*) reached its maximum content in the interaction of temperature-dominant multiple ecological factors with structural genes such as *CsANS* and *CsCHI* and transcription factors, furthermore producing the transition from phenolic acid to flavonoid biosynthetic pathways [57]. Light intensity influenced the content of three major metabolites, catechin, caffeine and theanine, through *NAC*, *WRKY* and *bHLH* family transcription factors and structural genes including *DFR* and *CHS* in different seasons, which inspired the regulation of metabolite content during different seasons to enhance quality [19]. Transcription and metabolism revealed that the higher accumulation of caffeine in tea plants than in coffee plants might be explained by the more copies of the *NMT* gene and its high expression level in tea plants; moreover, the caffeine biosynthesis process might be partially conserved [58]. Compared with green leaves in purple tea cultivar, the higher expression of transcription factors such as *NAC008* and *MYB23* and the accumulation in flavonoids such as copigmentation of quercetin might contribute to the formation of purple leaves [59]. The transcription factor *CsMYB90*, which was highly associated with anthocyanins such as cyanidin 3-O-galactoside, had been identified in the variety Zijuan and the overexpression was verified in tobacco. In addition, the cyanidin 3-O-galactoside glycosyl group, which affected anthocyanin accumulation, and the transcription factors *3T*, *3AT* and *ANS*, which were responsible for the regulation of anthocyanin content, were also identified in Zijuan [60,61]. Decreased chlorophyll might cause the occurrence of albinism in tea plants; RNA-seq and targeted metabolism uncovered that the absence of photosynthetic pigments activated their photoprotective mechanisms and that high nitrogen levels in albino tea plants facilitated the synthesis of photoprotective metabolites [62].

Cold stress among abiotic stresses was the main factor affecting the survival and breeding of tea plants, and substantial studies have been conducted regarding the regulatory mechanisms of cold tolerance in tea plants. Pyr/PYL-PP2C-SnRK2 in the ABA pathway enhanced the freezing tolerance through stomatal closure and performed the key role in signal transduction of freezing stress [63]. The difference in cold tolerance between tea tree shuchazao and yinghong9 varieties might be due to the activation of the CBF-COR pathway resulting from the high expression of amino acids [64]. Treatment with exogenous substance methyl jasmonate reduced the accumulation of ROS in tea plants under cold stress and maintained the stability of cell membranes to strengthen the cold tolerance of tea plants [64]. CsUGT91Q2 were identified as being involved in the regulation of cold tolerance in tea plants by glycosylating nerolidol to nerolidol glucoside [65]. The research on the regulatory mechanism of tea plants in response to cold stress and the excavation of cold resistance pathways will contribute to the elucidation of the cold resistance mechanism of tea plants and foster the breeding of cold resistant varieties. Apart from that, the planting pattern of intercropping tea plants with soybean crop, the withering process in production, the metabolite dynamics of fermentation process and shaking process, the variation of tea aroma in different seasons, different fertilization strategies, the difference of characteristic metabolites among different varieties, and other factors affecting metabolite changes and synthesis under various complex conditions and their roles on tea quality have tremendously driven the characterization research and variety breeding of tea plants [66,67,68,69,70,71,72], while providing novel ideas for future researchers to dig deeper according to the key pathways or regulatory networks of metabolite synthesis.

**Table 2:** Genes that have been cloned and characterized in tea plants.

Genes	Function Description	References
**Secondary metabolism**		
*CsAlaDC*	Theanine biosynthesis	[73]
*CsTS*	Theanine biosynthesis	[74]
*CsMYB5*	Regulating anthocyanin and proanthocyanidin biosynthesis	[75]
	Negatively regulating phenylpropane and shikimic pathway	
*CsMYB4a*	Regulating flavonoid 3′-hydroxylase	[76]
	Participating in flavonoid biosynthesis	
*CsF3′H*	Biosynthesis of terpenes in MVA pathway	[77]
*CsF3Ha*, *CsF3Hb*	Regulating Cinnamate 4-hydroxylase biosynthesis	[78]
*Cstps1*	Regulating of catechin production in phenylpropanoid pathway	[79]
	Regulation of epicatechin content	
*CsC4H*	Participating in flavan-3-ol biosynthesis	[80]
	Catalyzing caffeine biosynthesis pathway	
	Regulating Jasmonic acid biosynthesis	
*Cs4CL*		[81]
*CsANR*	Converting volatile compounds into β-primeverosides	[82]
*CsLAR*	Formation of the volatile component indole in oolong tea	[83]
*CsTCS*	Participating in aroma quality regulation	[84]
	Biosynthesis of linalool and nerolidol	
*CsAOC*	Regulating salicylic acid carboxyl methyltransferase and salicylic acid to produce methyl salicylate	[85]
**Aroma synthesis**	Insect-induced defense response	
*CsGT1*	Involved in herbivore defense	[86]
	Participating in the response of low temperature, high temperature, osmosis and hormone stress	
*CsTSA*, *CsTSB2*	Respond to abiotic stress	[87]
	Response to most abiotic stresses (including salinity, heavy metal toxicity, drought, high temperature and phytohormones)	
*CsTPS08*, *CsTPS10*	Participating in pest defense	[88]
	Significantly improving the cold tolerance of plant	
*CsLIS/NES*	Exogenous application of SPM to improve tea plants drought tolerance	[89]
*CsSAMT*	Low temperature induced during dormancy	[90]
	Directing the gene expression of tea somatic embryo nucleus	
	Regulating the absorption of ammonium from soil by tea plants roots	
**Abiotic and biotic stresses**		
*Cstps1*	Regulating nitrogen absorption by tea plants root system	[79]
*CsCPI3*	Regulating tea plants dormancy	[91]
*CsVQ*		[92]
*CsAQP*		[93]
*CsGPX2*		[94]
*CsTPS08*, *CsTPS10*		[88]
*CsPPO*		[95]
*CsSPMS*		[96]
*CsTUA*		[97]
**Others**		
*CsH1*		[98]
*CsAMT1.2*		[99]
*CsNRT2.4*		[100]
*CsLAX2*		[101]

### 3.3. Whole Genome Resequencing

Whole genome resequencing enabled research at the population level to analyze the mechanisms of adaptive evolution and varietal improvement in tea plants. In recent years, the massive number of resequencing data have accelerated the genetic breeding process in tea plants. Genome resequencing had screened for *CsMYB1*, which existed only in modern tea cultivars, as the regulator of trichome and galloylated cis-catechins biosynthesis and had been targeted in domestication to enhance tea plants’ flavor [102]. Genome resequencing of 120 ancient tea plants species from eight different taxa unearthed candidate genes significantly associated with leaf traits (*TEA012477*, *TEA028016*, *TEA025567*, *TEA017338*) and plant types (*TEA029928* and *TEA012294*), the results of which can be applied to genetic improvement and functional excavation of tea plants [103]. Cultivated germplasm was divided into CSS and CSA subgroups by resequencing the whole genomes of 30 cultivated and three wild species, while the CSS subgroup was revealed to possess more genetic diversity. The variation and environmentally adapted selective regions identified in the research could be adapted for subsequent molecular marker breeding or fingerprinting [104]. The abundant SNP and insertion deletion genetic variants loci were identified in the resequencing data of Jinxuan and other 98 tea varieties, the high-density genetic map was constructed and 25 representative markers associated with leaf area were exploited; the work laid the foundation for quantitative trait localization and breeding of superior varieties of tea plants [21]. In total, 139 tea plants resources from around the world were utilized to explore the evolution of germplasm, where hybridization increased heterozygosity between tea plants populations together and population genetic results indicated that the domesticated CSS had superior disease resistance and flavor than CSA [50]. Since tea germplasm resources of different varieties had significant trait differences and genetic variation among varieties, future resequencing of larger samples was thus the necessary direction for tea plants breeding works.

## 4. Molecular Markers

DNA-based molecular markers were genetic markers that offered the advantages of genetic stability, polymorphism, speed and convenience and were widespread techniques applied in crop breeding improvement [105,106]. The development of molecular markers had also facilitated research on the origin and evolution of tea plants, breeding and refinement processes. So far, various markers have been developed and applied in tea plants, such as random amplified polymorphic DNA (RAPD) and inter simple sequence repeat (ISSR) markers to analyze the degree of genetic diversity and population genetic structure in tea clones and to select superior varieties for the further hybrid breeding materials of tea plants [107]. The development of expressed sequence tag-simple sequence repeat (EST-SSR) markers in tea oil plants could be applied for the identification of tea oil varieties and protection of genetic resources [108]. SNP markers transformed into competitive allele-specific PCR (KASP) were found to be associated with nitrogen accumulation and biomass in tea plants, and functional markers could be utilized in the future to produce high nitrogen varieties [109]. A genetic linkage map based on amplified fragment-length polymorphism (AFLP), simple sequence repeat (SSR) and RAPD markers was constructed to design the molecular markers related to tea plants resistance and quality, as well as to promote the localization and mining of quantitative trait loci (QTLs) for essential traits in tea plants [110]. In addition, intron length polymorphic (ILP), sequence-related amplified polymorphism (SRAP), start codon targeted polymorphism (SCoT), cleaved amplified polymorphic sequence (CAPS) and other markers have also been developed and applied in tea plants, which laid the foundation for subsequent marker-assisted breeding and genetic diversity analysis [23,111,112].

Given the prominent role of SNPs and SSRs in plant breeding research, they were also widely available in tea plants. Genetic relationship and population structure analysis of 140 varieties of oil tea plants based on SSR markers reflect the genetic relationship between different germplasm resources, and they could serve for variety identification, classification and kinship analysis of oil tea plants [113]. The SSR markers identified from the transcriptome associated with tea-specific traits were linked to their transcription factors, which revealed the interaction with tea resistance and quality, and the developed transcription factors and SSR combined markers have potential applications in tea trait and marker association breeding [114]. The whole genome of shuchazao was identified by SSR markers, and the linkage map of the Zhuyeqi and Yunkang 10 population was constructed. The phylogenetic analysis revealed that the results were almost consistent with its genetic background, and the SSR markers developed were highly polymorphic, which had certain significance for parsing the historical issues of evolution or domestication [115]. Through the analysis of 142 tea cultivars with SNP loci, which indicated that Yunnan Province was the main area of origin and domestication of CSA, while CSSs were distributed in Eastern China, eight markers were developed that could identify germplasm and have been deployed for the construction of DNA fingerprinting [116]. In addition, SNP variant loci obtained from whole genome resequencing of 96 F1 hybrid progeny were sequenced to construct the high-quality genetic map, where insertion loci located at potential QTL loci could be distinguished between large and small leaf tea plants [21]. SNP markers were also applied to distinguish Oolong tea from other cultivars. In total, 75 SNP loci were generated for genotyping in 100 Oolong tea cultivars, while DNA fingerprinting was constructed for four major Oolong tea producing regions based on SNP locus information [117]. Genetic mapping of the SNP markers identified in the F1 population of Longjing43 crossed with Baihaozao was constructed and QTL analysis revealed the loci associated with flavonoid content [118]. Furthermore, key genes related to characteristic metabolites such as theanine were identified in SNP markers generated from the population consisting of 191 germplasms, while 17 SNP markers related to specific metabolites were determined in another 98 germplasms, providing reliable marker information for the research of tea metabolites [20]. Six key genes associated with leaf traits were obtained after GWAS of 338 tea germplasm mined for SNP loci verified by linkage disequilibrium, which provided the essential reference for subsequent breeding of leaf traits [119].

## 5. Major Directions and Measures for Tea Plants Breeding

Nowadays, improving current crops or developing new crops with high yields, excellent quality and low costs, abiotic and biotic stress resistance were the current foremost directions for breeders [11,120,121,122,123]. Food crops, including wheat, maize and rice, were modified to increase yields and nutrition to meet the needs of the growing population [121,124,125]. However, as the economic crop that is consumed on a regular basis and whose product, tea, has health benefits, the promotion of high yield, quality and resistance is the leading concern in tea plants breeding today; hence, this section presented existing initiatives in tea breeding with the aim to provide references to researchers.

### 5.1. High Productivity

Acidic soils could restrict the yield of most crops, and Al^3+^ ions in the soil may suppress the development of the root system [126]. However, Al is a special element for tea plants due to the fact that tea plants were well-adapted to grow in an acidic environment. The appropriate soil pH for tea plants was 4.5–5.5, yet 46% of soils nationwide contained pH < 4.5 [127]. In previous hydroponic experiments, the presence of 50 μM Al maximized the growth of tea branches and 300 μM Al tripled the root biomass [128]. The certain range of Al^3+^ caused remarkable growth and physicochemical improvements in different tea varieties [129]. The application of shellfish amendments in acidified tea plantations could contribute to a certain degree to the yield, quality and economic value of tea leaves [130]. Although the understanding of tea plants under the influence of Al was still extremely limited, breeders were now further dissecting the mechanisms of Al tolerance and utilization in tea plants, thus setting the foundation for optimizing acidic tea plantations, optimizing the yield quality of tea plants and breeding acid-tolerant crops for the future [131]. Phytohormones were endogenous signaling molecules that were necessary for the development of tea plants [132]. The main seven hormones were described for their application in tea plants. Indole-3-butyric acid (IBA) and N-1-naphthylphthalamic acid (NPA)-treated tea plants generated more lateral roots, and the growth of lateral roots was stimulated by growth hormone synthesis and accumulation at low nitrogen concentrations [133]. The cloned growth hormone transporter gene CsLAX2 could regulate tea plants dormancy and confirmed that growth hormone exerts a vital role in tea plants dormancy [101]. Ethylene was a gaseous hormone whose signaling was involved in regulating the accumulation of secondary metabolites and could increase catechin content [134]. Application of exogenous ethylene glycol effectively inhibited flowering of tea plants and improved tea bud growth and yield [135]. Salicylic acid was an immune-related hormone [136] that inhibited phenylpropane and flavonoid metabolic pathways after salicylic acid treatment, increasing lignin content and defense and disease resistance, and indirectly for yield maintenance or enhancement [137]. Tea plant varieties with higher salicylic acid content were able to enhance the response to anthracnose infection with wilt and attenuated pests and diseases [138,139]. Jasmonic acid likewise coordinated plant defense against pests and pathogens, with jasmonic acid regulating Polyphenol oxidases’ (PPOs’) defense against tea geometrids [140]. Brassinosteroids (BRs) were ubiquitous phytosterols that responded to multiple abiotic stresses [141]; besides, exogenous application increased sucrose, starch and flavonoid content to promote tea plants’ growth and development [142]. Melatonin could promote photosynthesis and biomass accumulation in tea plants and could alter polyphenol and caffeine content to improve quality [143]. Exogenous melatonin treatment enhanced terpene biosynthesis and promoted growth of tea plants [144]. The exogenous implementation of gibberellin, a plant hormone that promoted germination and growth [145,146], facilitated the sprouting of tea buds and the development of branches, and increases yields by half [147]. The yield of tea plants was enhanced at 2 g per acre of chitosan, which induced significant up-regulation of amino acid metabolism and carbohydrate metabolic pathways in tea plants [148]. Pruning of summer oolong tea at 30 cm promoted the growth of lateral branches, while reducing the ratio of total polyphenols to free amino acids to improve quality [149]. The application of nitrogen, phosphorus, potash and biochar-based fertilizers to tea plants had been demonstrated to achieve improved yield and quality, but the specific application should also take into account the disparities brought about by climate, altitude, agronomic practices, soil condition and variety [150,151,152,153,154]. Tea microbes contributed to improved growth, quality and yield of tea leaves. For example, tea seedlings inoculated with arbuscular mycorrhizal fungi (AMF) in pot experiments increased stem biomass and leaves area, and higher soluble sugar and protein content [155]. PGPR strains *Enterobacter lignolyticus*, *Bacillus pseudomycoides* and other rhizobacteria were used to promote tea plants growth [156]. Endophytic microbiota could also promote tea plants growth and the production of theanine secondary metabolites [157]. Growth was facilitated by the production of volatiles such as terpens by tea plants’ inter-rooted bacteria, as well as the induction of indoleacetic acid production and phosphate solubilisation [158]. Altogether, the tea microbiome supported nutrient uptake, quality improvement and stress mitigation, whilst utilizing natural resources for yield enhancement remained a sustainable research topic.

### 5.2. High Quality

**Figure 2:**
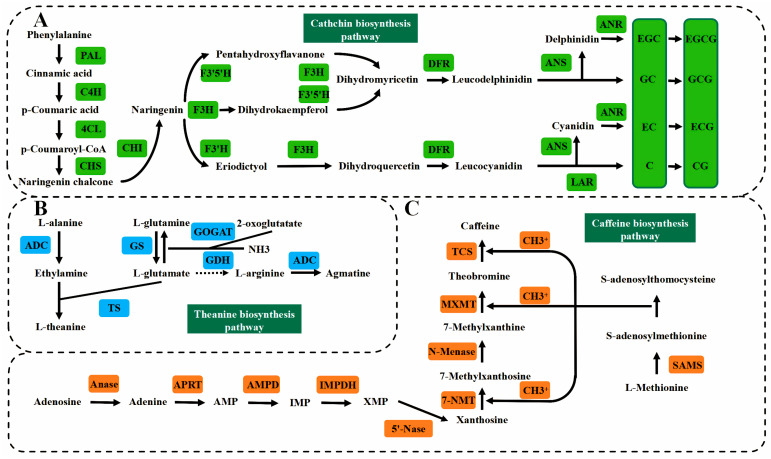
Diagram of genes and metabolite pathways associated with the major secondary metabolites of tea plants. (**A**) Catechin biosynthetic pathway. (**B**) Theanine biosynthesis pathway. (**C**) Caffeine biosynthesis pathway.

Tea polyphenols, catechins, caffeine, amino acids and more are the main secondary metabolites in tea, and their levels are the main indicators of the flavor quality of tea, whose biosynthetic pathways have been characterized (Figure 2); thus, conducting research on the molecules and mechanisms affecting these characteristic compounds will contribute to the improvement of tea quality. Catechins, caffeine and polyphenols were the key determinants of the bitterness and astringency of tea leaves [159,160,161]. Tea plants included six major catechin types, which were (+)-catechin (C), (−)-epicatechin (EC), (+)-gallocatechin (GC), (−)-epigallocatechin (EGC), (−)-epicatechin-3-gallate (ECG) and (−)-epigallocatechin-3-gallate (EGCG), respectively. Only EGCG and ECG were galloylated, accounting for about 75% in total catechins and they contributed more towards flavor than the nongalloylated catechins [162]. Caffeine was a purine alkaloid, accounting for more than 95 per cent of the total alkaloids in tea plants. The caffeine content in tea was affected by the developmental stage and treatment, and it varied greatly in different parts, with the content in the leaves being higher than in the stems. It also accumulated more in the spring than in summer [6,163]. Theanine, which accounted for 60% of the amino acids in tea, was the main contributor to the freshness and sweetness of the tea leaves [164,165]. Theanine was naturally occurring in abundance as a free nitrogen compound, and as a non-protein amino acid was synthesized from ethylamine and glutamic acid by theanine synthase, with synthesis occurring preferentially in the roots, which was then transported to the new shoots [166,167]. The theanine metabolic pathway was divided into synthesis in the roots and hydrolysis in the buds, where the amino acids and proteins degraded by hydrolysis enzymes were used for the growth of tea buds [168]. The amount of theanine content could be influenced by different varieties, which meant that different genetic factors determined the level of theanine [20]. In addition to this, its content shifts with the seasons, with higher levels in spring tea than in summer and autumn [169]. Disturbance by abiotic and biotic stresses could reduce the accumulation of theanine in different parts [170,171]. In addition, genes associated with secondary metabolites were enumerated to understand the synthesis pathways and influencing factors better (Table 3). In modern quality breeding, diverse methods and techniques regarding exogenous applied substances, various stages, temperature, genetic engineering and metabolomics represented by LC-MS were used to solve the stumbling blocks of tea in quality enhancement.

The tea tree was a plant readily enriched in selenium, and exogenous spraying of selenium nanoparticles markedly improved the quality of summer tea, enhanced the defenses of the tea plants, visibly altered the ratio of compounds such as tea polyphenols, improved sweet freshness and reduced bitterness, optimized the bright spot for the application of nanomaterials in tea plants [172,173]. Foliar application of glycine-chelated sodium selenite and zinc sulfate heptahydrate could achieve selenium enrichment in tea leaves and indirectly increased the economic value of tea by extending the harvesting time [174]. Certain ranges of fertilizers could promote plant development, and magnesium supplementation in hydroponic experiments could increase the amino acid content in tea plants at low nitrogen levels and complete the fertilization strategies for tea plantation management [175]. The decline of tea quality might relate to the soil environment, long-term application of organic fertilizer can improve the acid soil and the yield, quality and performance, while continuous application of chemical fertilizer will further worsen the soil and aggravate the acidity, which will hinder the growth of tea plants [176]. Research on tea plants has often focused attention towards the growing period, while foliar nitrogen application during the winter dormancy of tea plants improves the nitrogen content of mature leaves with the quality and yield of spring tea, while fertilizer dosage can be reduced [177]. The interaction between hormones and metabolites within the tea plants also influenced quality, as the expression of *CsJAZ6* in tea plants inhibited the JA pathway, which regulated most secondary metabolites, notably catechins, and the interaction between *CsJAZ6* and catechin biosynthesis regulators negatively regulated catechin accumulation [178]. Apart from endogenous hormones, endogenous hormone signaling factors could also mediate the synthetic pathways of caffeine, EGCG and theanine to respond to temperature and improve the quality of tea leaves [179]. The diurnal temperature difference at 10 °C clearly affected primary and secondary metabolites, with the lowest levels of polyphenols and catechins and higher levels of amino acid and theanine synthase expression, but the actual application on a certain scale was still constrained by the environment and climate [180]. Different pruning processes also affect the quality of tea leaves, and in Yunnan tea plantations, metabolite data from the unpruned tea (UPTT) revealed that levels of amino acids leading to sweetness increased, while levels of bitter and astringent catechins and caffeine decreased [181]. The plant-growth regulator treatment of tea guava tissues located in cell suspension system subserved the accumulation of tea polyphenols and offered support for application in practical tea garden cultivation [182]. Variable degrees of shade treatment also altered the quality of the tea leaves, with tea plants cultivated under shade not only having elevated theanine and caffeine content and reduced polyphenol content affecting taste, but also stimulating the biosynthesis and transport of theanine at diverse sites [183,184]. With the exception of external factors such as environmental disturbances, the age of the tree was also the factor that impacted its quality, the aroma of tea leaves of various ages gradually increased with age to render them lighter, fresher and sweeter, and the flavonoids that caused tea leaves to be bitter will lessen [185]. Furthermore, HPLC-MS, GC-MS and LC-MS techniques have been widely applied to metabolomic studies to determine the synthetic pathways and changes of various metabolites to resolve the mechanisms affecting the quality of tea leaves [67,186,187,188,189,190].

Moreover, the aroma of tea was also crucial to the quality of tea, which was mainly affected by the nature of the tea plant on its own, the production process, and many factors in its formation and release [191]. While non-volatile compounds mainly contributed to the flavor and mouthfeel of tea, volatile aroma compounds were the basis of tea aroma [192]. The main volatile aroma compounds in tea leaves were terpenes, alcohols, aldehydes and ketones, which performed the key role despite representing only a very low percentage of the dry weight of the tea leaves [193]. Aroma compounds in tea leaves were mostly derived from volatile fatty acid derivatives (VFADs), volatile terpenoids (VTs) and volatile phenylpropanoids/benzenes (VPBs) [194]. Among them, biosynthetic genes associated with tea aroma formation have been reported and part of them have been characterized in vitro [195]. Besides, the dynamics of volatile and non-volatile compounds in different states of black, white, yellow, green and oolong teas have been characterized to enhance the aroma [196,197,198,199,200]. Interestingly, volatile and non-volatile phenylpropanoids/benzoflavonoids in tea shared a common upstream synthetic pathway, with phenylalanine entering the phenylalanine metabolic and synthetic pathways, respectively. The former continued to form volatile BPs and the latter formed flavonoids, including anthocyanins [201]. This pathway associated both volatile and non-volatile synthetic pathways that could influence the flavor and aroma of tea, which might support the design of the new tea germplasm to improve the quality of tea.

**Table 3:** Information on main metabolite genes in tea plants.

Metabolite	Genes	Biosynthetic Pathway	References
L-theanine	*TS GS ADC GOGAT GDH*	Nitrogen metabolism pathway	[202,203]
Galloylated catechins	*ECGT UGGT*	Shikimate pathway	[204,205]
		phenylpropane pathway	
Caffeine	*SAM TCS MXMT*	S-Adenosyl-L-methionine (SAM) pathway, nicotinamide adenine dinucleotide pathway,	[84]
		adenosine 5-monophosphate pathway, guanosine 5-monophosphate pathway	
(E)-Nerolidol	*LIS/NES*	Terpene synthesis pathway	[89]
Linalool	*LIS/NES*	Terpene synthesis pathway	[89]
α-Farnesene	*AFS*	Terpene synthesis pathway	[206]
Anthocyanin	*PAL C4H 4CL CHS*	Phenylpropane pathway,	[207]
	*CHI F3′H F3′5′H*	flavonoid pathway	
	*F3H DFR ANS LAR*		
Indole	*CsTSA CsTSB2*	Shikimic acid-derived pathway	[87]

### 5.3. High Resistance

**Figure 3:**
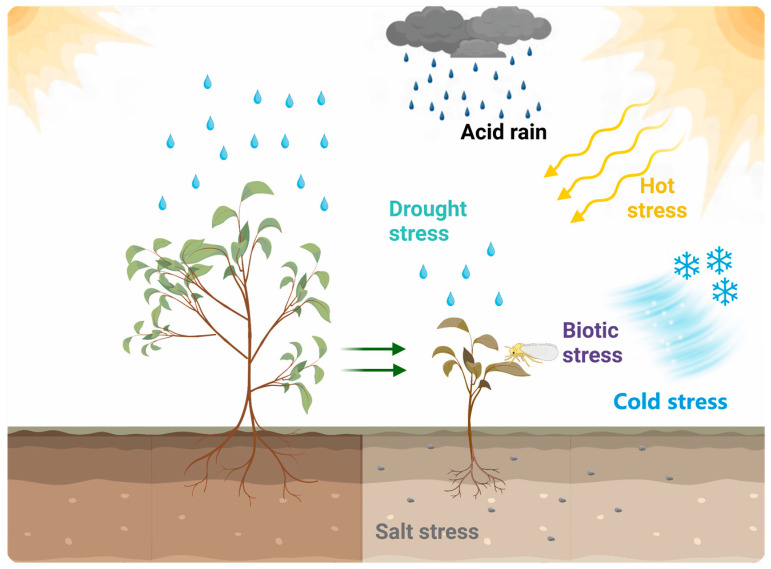
Major biotic and abiotic stresses exposed to tea plants. lmage was created with the tools of BioRender (https://app.biorender.com/ (accessed on 18 June 2023)) and Figdraw (https://www.figdraw.com/ (accessed on 18 June 2023)).

Biotic and abiotic stresses have been the cause of challenges for most crops, including tea plants (Figure 3) [208,209,210]. To alleviate the hazards associated with stresses such as acid rain, low temperature, drought, pests and diseases, salinity and heat, which were common in tea plants, breeding techniques such as omics, molecular experiments and marker-assisted breeding were implemented to address these situations. Due to the instability of the current climate, tea regions in southern China were mostly affected by acid rain. When the pH of simulated acid rain reached 2.5 or 3.5, the photosynthesis of tea plants was restricted and the metabolic pathways were affected, damaging the development of tea plants, whereas acid rain with a pH of 4.5 had no negative impact on tea plants [211]. At the simulated acid rain pH level of 2.5, the reduction in chloroplast numbers and stomatal density were observed by transmission electron microscopy, and the expression of several genes related to photosynthesis and carbohydrate metabolism was altered; in addition, the plant hormone signal transduction pathway was affected by the high acidity of acid rain [18]. Acid rain not only affected the yield of tea, but also threatened the quality and safety of tea leaves. Therefore, the mechanism and resistance measures of tea plants to acid rain requires further parsing and exploration.

For regions such as northern China with high latitudes, massive numbers of tea plants might die during overwintering caused by low temperature or freezing stress. Therefore, plenty of studies have been focused on breeding for cold tolerance in tea plants, such as the role of exogenous substances application, response gene mining and transcriptional regulation mechanisms on tea tree breeding. The treatment of exogenous selenium stabilized the photosynthetic system and membrane stability and improved the cold tolerance of the tea plants, while the oxidation products of tea polyphenols were reduced and metabolites that contributed to quality such as sugars and theanine were increased [212]. The application of exogenous jasmonic acid scavenged reactive oxygen species and maintained membrane stability under cold stress, while the *CsMYB* transcription factor functioned not only as the key component of the signaling response, but also as the binding point between cold stress and signal transduction [213]. Exogenous application of 5-aminolevulinic acid (ALA) increased catechin and procyanidin B2 content and altered carbohydrate and flavonoid content to enhance cold tolerance [214]. Exogenous melatonin treatment alleviated cold-induced photosynthetic damage by enhancing antioxidant defense and reducing oxidative stress, but the molecular mechanism of cold tolerance has not been explored [215]. The plant tended to increase its freezing tolerance after a period of exposure to cold environments [216], and similarly, tea plants also have to undergo cold domestication to increase their cold resistance, and their domestication process triggered back a series of regulatory networks, such as carbohydrate metabolism and calcium signaling pathways, whose response profiles in domestication have also been revealed [217]. The genes *CsCBF5*, *CsCBF3*, *CsFAD2* and *CsFAD5* were detected as exerting an essential role in response to cold stress, providing ideas for subsequent molecular breeding with potential value [218,219]. Furthermore, chromosome accessibility, circular RNA and genome resequencing excavated potential value for cold tolerance in tea plants from different research perspectives. Although these measures provide insights in the cold tolerance of tea plants, the mechanisms of cold tolerance require further exploration and validation.

Drought stress could shrink the buds and leaves of tea plants and seriously affect the yield and quality of tea leaves. For this reason, researchers have conducted studies on the drought tolerance mechanisms of tea plants. By developing hyperspectral imaging techniques to simulate physiological data such as malondialdehyde and electrolyte leakage in tea plants, researchers could obtain the degree of damage to tea plants under drought stress and take prompt measures to reduce losses based on the feedback results [220]. The storage of K^+^ by mesophyll cells was a vital process for drought resistance in tea plants, and furthermore, treatment with exogenous K^+^ (5 mM) was able to alleviate damage under drought stress [221]. Fluvic acid at 0.1 mg/L not only regulated sucrose and starch metabolism [222] but also enhanced ascorbic acid metabolism and flavonoid biosynthesis to protect against drought stress [223]. The application of calcium nitrate in the soil resulted in increased cytosol pH and raised amino acid levels such as tyrosine in tea plants under drought stress, and simultaneously activated the abscisic and jasmonic acid pathways to participate in stomatal regulation [224]. Fatty acid unsaturation and H^+^-ATPase activity were improved and maintained by the application of the exogenous substance 0.2 mM spermidine (Spd) or spermine (Spm), thus alleviating the damage caused by drought stress in tea plants [225]. Eugenol modulates the expression of an uridine diphosphate (UDP)-glucosyltransferase, UGT71A59, which altered the steady state of abscisic acid and regulated stomatal closure to promote drought tolerance in tea [226]. Besides, 24-epibrassinolide (EBR) also promoted stomatal closure to resist stress by regulating the expression of stomatal-related genes [227]. Seventy-six *CsbZIP* genes were identified in the tea genome, and then combined with 13 ABFs in abscisic acid signaling to screen for *CsABF2*, *CsABF8* and *CsABF11*, transcription factors that could be applied in subsequent studies to regulate drought resistance [228].

Pests and diseases in tea plants were also the primary factors that reduced yields and affected the quality of tea plants. Anthracnose, caused by *Colletotrichum*, proved to be one of the most serious diseases affecting tea plants, with infected leaves often suffering from water-soaked disease and lesions to the point of necrosis, which largely affected the yield of tea plants [229]. Tea green leafhoppers, tea geometrids and tea aphids were the main pests of tea plants [230]. (E)-Nerolidol enhanced tea tree defenses and protected tea plants from pests such as *Colletotrichum* by inducing the production of defense-related compounds to accumulate in the plant [231]. (Z)-3-Hexenol operated as a potential regulator, activating JA signaling and reinforcing the resistance of tea against tea geometrid [232]. Early defense signaling, JA biosynthesis and substances associated with defense responses were intensified and accumulated by indole following enhanced Ca^2+^ signaling, thus supporting tea plants to resist pests [233]. *CsTCP10* was a positive regulator of tea plants resistance to Gray blight disease and molecular experiments showed that miR319a inhibited the expression of *CsTCP10*, thereby reducing resistance in tea plants [234]. In the event of pests and diseases in tea gardens, the large-scale application of chemicals and pesticides performed a crucial role in fighting off pests and diseases but came at a tremendous cost to the environment. Environmentally friendly methods such as microbial intercropping or biocontrol were expected to have a dampening effect on pests and diseases without damaging the environment [235].

Tea plants were suitable for growing in acidic soils, whereas saline soils affect the uptake of ions and water from the roots and thus suppressed the growth of tea plants. Genes associated with theanine biosynthesis, including *CsGOGATs*, *CsAlaDC* and *CsTSI*, were induced to be significantly up-regulated under salt stress, and exogenous administration of theanine improved tolerance by increasing the activity of antioxidant enzymes such as SOD [236]. Tea plants inoculated with Arbuscular mycorrhizal fungi (AMF) alleviated salt stress through osmoregulation [237]. Under AMF (*Glomus etunicatum*) treatment, the amino acid content of salt-stressed tea leaves was elevated, and the expression of resistance-related genes *CsTCS1*, *CsAPX* and *CsHMGR* was increased, as were the activities of antioxidant enzymes [238]. Long non-coding RNAs (lncRNAs) excavated from tea genome were involved in GOLS and calcium signaling pathways to cope with salt stress, in which lncRNA *MSTRG.139242.1* and *TEA027212.1* interacted to respond to stress [239]. Various *CsHsf* in tea plants were involved in diverse stress responses, and the identification of *CsHsfA2* increased the heat tolerance in transgenic yeast, underpinning the genetic engineering breeding [240]. Exogenous 24-epibrassinolide (BR) not only alleviated the stress caused by high temperatures, but also induced GS and GOGAT activity to increase the theanine content in the tea leaves, thus improving the quality of the tea leaves [241]. Overexpression of *CsCDPK20* and *CsCDPK26* in transgenic *Arabidopsis thaliana* increased their heat tolerance and presumably positively regulated heat tolerance in tea plants [242]. Heat shock proteins *CsHSP90* and *CsHSP17.2* perform key roles in heat stress in tea plants [243,244]. However, relatively little research has been conducted regarding the heat tolerance of tea plants, and the deeper molecular level of research requires further investigation.

## 6. Conclusions and Prospects

**Figure 4:**
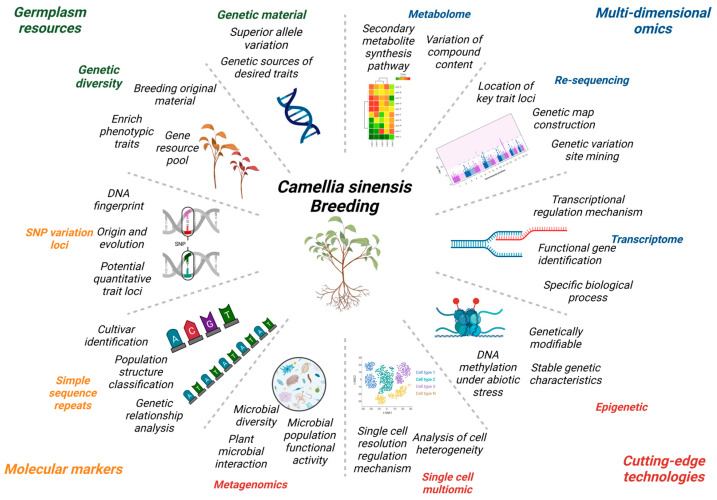
Diagram of the role or contribution of multiple perspective-based techniques to tea plants breeding. lmage was created with the tools of BioRender (https://app.biorender.com/ (accessed on 28 June 2023)).

In this study, the current status and utilization of tea plants’ germplasm resources were summarized, the applications of multi-dimensional omics in tea plants were presented, and existing strategies for the current key breeding directions and prospects in tea plants were also proposed (Figure 4).

**Figure 5:**
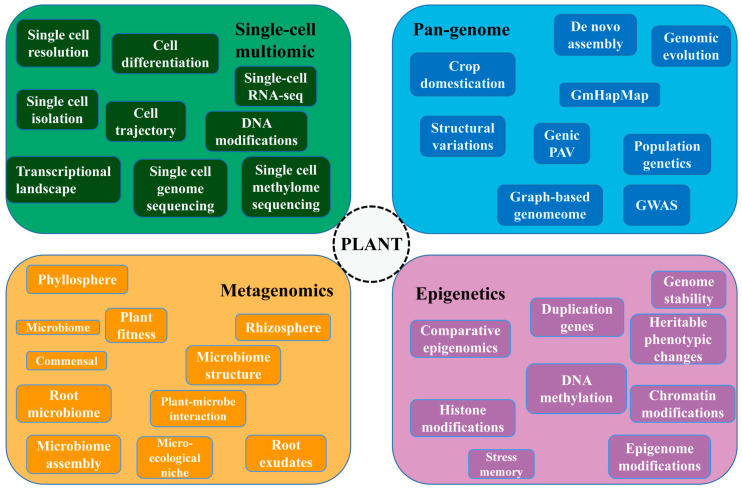
Schematic representation of key search terms in different cutting-edge technologies.

For further exploring the genetic breeding rules of tea plants, breeding enriched varieties that satisfy high yield quality and resistance and solve the problem of prolonged breeding cycles and technological innovation in breeding. Therefore, we presented the most popular and cutting-edge technologies and frequently searched terms in the field of plant for the reference of researchers and aimed to pioneer novel breeding ideas and directions for researchers (Figure 5).

### 6.1. Single Cell Multiomic

With the advancement of next-generation sequencing technologies, various multi-omics approaches, including transcriptomics and genomics, have been widely used to address biological inquiries and unravel underlying mechanisms at the multicellular level. However, in recent years, single-cell sequencing has emerged as a powerful technique, enabling researchers to delve into the intricacies of transcription and biological processes at the individual cell level. This single-cell transcriptome sequencing approach offered a unique opportunity to explore the complexity and heterogeneity of cellular processes with high resolution [245,246,247]. The potential of single-cell transcriptomics was demonstrated in the model crop *Arabidopsis thaliana*, where sequencing solved the complex biological processes undergone by *Arabidopsis* roots during their transition from stem cells to differentiation [248,249]. With the advantages of sequencing, it was widespread in crops such as maize (*Zea mays*), rice (*Oryza sativa*) and tomato (*Solanum lycopersicum*) [250,251,252,253,254]. In the context of tea plants, single-cell transcriptomics mapped the developmental trajectory of leaf cells and revealed new pathways related to catechin ester metabolism, providing fresh insights into the synthesis of secondary metabolites [255]. Single-cell sequencing initially emerged and was applied in animal and human cells. So far, single-cell transcriptome sequencing has still been at the early stage in the plant kingdom. Some issues such as little information about cell types or difficulties in releasing cells have still been outstanding and unexplored in numerous plant species, presenting both opportunities and challenges [256,257,258,259]. Future advancements in technologies, such as single-cell metabolomics, single-cell genomics and single-cell methylation, were expected to address the complex issues related to cellular composition, interaction mechanisms and functional characteristics in the plant with inferior genetic characteristics like tea plants [260,261].

### 6.2. Pan-Genome

High-quality reference genomes delivered knowledge of the genetic characteristics of plants, particularly in the context of domestication breeding and basic plant research. Pan-genomes assembled from genome sequences of multiple species could carry information on the genetic diversity of a species. They were recognized as a novel reference tool, serving as a valuable resource for crop breeding enhancement, as well as for evolutionary and functional genomics research [262,263,264,265].

The integration of wild and cultivated sorghum pangenomes generated information on genetic variation that could not be captured by a single reference genome, which contributed to phenotypic characterization and facilitated sorghum improvement [266]. Similarly, the pangenomes of 118 wild and cultivated peas complemented the multitude of genes and sequence gaps that were missing from the reference genome. This broadened our understanding of the genetic background on peas, shedding light on their domestication and evolution [267]. Additionally, the construction of the soybean pangenome led to the interlinking of genes that determine essential traits with newly uncovered genetic variants, favoring the resolution of current genetic bottlenecks [268,269]. Otherwise, the assembly of pangenomes of grains, vegetables and fruits such as radish (*Raphanus*), cucumber, mung bean (*Vigna radiata* L.), cotton and strawberry were used for biological research and genetic breeding [270,271,272,273,274]. Until now, several high-quality chromosome-level reference genomes have also been assembled in tea plants, and we firmly believe that more genomes will be produced in the future, while the emergence of tea plants pangenomes was expected to represent the new reference for tea plants’ genetic research and breeding in the future.

### 6.3. Metagenomics

Microorganisms were resident throughout the ecosystem and strong interactions exist between plants and various microorganisms. Today, rather than traditional research methods, macrogenomes could capture the genetic information of fungi and bacteria, thus revealing the mechanisms of plant–microbe interactions and regulation, and guaranteeing more sustainable breeding strategies [275,276,277]. Relatively more research was focused on inter-rooted microorganisms that lived in the soil than on other microorganisms. Inter-root microbes often modulated plant development by facilitating nutrient uptake and stimulating resistance to biotic and abiotic stresses through nitrogen fixation, phosphate solubilisation [278,279,280]. Plants recruited microbiota performing specific functions to satisfy their developmental demands through characteristics such as their own secretions and immune systems [281].

Tomatoes under salt stress could be protected from salinity by a synthetic bacterial community of the desert plant *Indigofera argentea* [282]. The existence of the bacterium *Brevibacterium* linens RS16 restored photosynthetic properties and elevated salt tolerance in rice under salt stress [283]. Analysis of the microbial community of diverse resistant tomato varieties detected and cultured *Xanthobacteria Xanthobacteria* resistant to wilt caused by the *Ralstonia solanacearum* pathogen, and this study elucidated the mechanism by which the microbiota could establish protection against the pathogen in plant [284]. Selection and cultivation of microbiota for plant breeding and the utilization of the positive effects of advantageous microorganisms for the welfare of plant was the forward-looking approach to the use of microbial action for plant reclamation. Extensive studies were conducted on the regulation of plant–microbe interactions [285] and the application of this model to tea plants might lead to the search for potential microbial groups that contributed to the high yield and resistance of tea plants.

### 6.4. Epigenetics

Epigenetics received widespread attention in the field of plant genetics, which referred to heritable modifications of gene function that did not involve DNA sequence alterations [286,287]. Epigenetics comprised DNA methylation, histone modification and RNA interference, which modified the stress response of plant under different stress environments [288], while DNA methylation was one of the most passionately studied epigenetic mechanisms that could manipulate gene expression, genome stability and chromatin structure [289,290]. Up till now, DNA methylation has been deployed in plants such as maize (*Zea mays* L.), tomato (*Solanum lycopersicum* L.), rice *(Oryza sativa* L.), potato (*Solanum tuberosum* L.), citrus and land cotton (*Gossypium hirsutum* L.) to investigate various biological events [291,292,293,294,295,296]. Pronounced changes in the transcript levels of cytosine-5 DNA methyltransferase and DNA demethylase in tea plants demonstrated the pivotal role of DNA methylation in the regulation of abiotic stresses and had served as a reference for the analysis of epigenetic mechanisms in tea plants [297]. Epigenetics was the prospective area to exploit the ability of DNA methylation for stable transmission of genetic traits to nurture and maintained the superior characteristics of tea plants in the future.

## Figures and Tables

**Table 1:** Published information of tea genomes.

Cultivar	Species	Genome Size/Gb	Reference
Yunkang10	*C.sinensis* var. *assamica*	3.02	[48]
Shuchazao	*C.sinensis* var. *sinensis*	3.14	[49]
Shuchazao	*C.sinensis* var. *sinensis*	2.94	[22]
Shuchazao	*C.sinensis* var. *sinensis*	3.20	[51]
DASZ	Wild tea tree	3.11	[52]
Biyun	*C.sinensis* var. *sinensis*	2.92	[53]
Longjing43	*C.sinensis* var. *sinensis*	3.26	[50]
Tieguanyin	*C.sinensis* var. *sinensis*	3.06	[54]
Huangdan	*C.sinensis* var. *sinensis*	2.94	[37]
Huangdan	*C.sinensis* var. *sinensis*	5.53	[37]
Duyunmaojian	*C.sinensis* var. sinensis	2.97	[55]
Camellia	*C.oleifera Abel.* var “Nanyongensis” *oleifera*	2.89	[56]

## Data Availability

Data are contained within the review.
